# Finding phrases: On the role of co-verbal facial information in learning word order in infancy

**DOI:** 10.1371/journal.pone.0224786

**Published:** 2019-11-11

**Authors:** Irene de la Cruz-Pavía, Judit Gervain, Eric Vatikiotis-Bateson, Janet F. Werker

**Affiliations:** 1 Integrative Neuroscience and Cognition Center (INCC–UMR 8002), Université Paris Descartes (Sorbonne Paris Cité), Paris, France; 2 Integrative Neuroscience and Cognition Center (INCC–UMR 8002), CNRS, Paris, France; 3 Department of Psychology, University of British Columbia, Vancouver, British Columbia, Canada; 4 Department of Linguistics, University of British Columbia, Vancouver, British Columbia, Canada; University of the Basque Country, SPAIN

## Abstract

The input contains perceptually available cues, which might allow young infants to discover abstract properties of the target language. Thus, word frequency and prosodic prominence correlate systematically with basic word order in natural languages. Prelexical infants are sensitive to these frequency-based and prosodic cues, and use them to parse new input into phrases that follow the order characteristic of their native languages. Importantly, young infants readily integrate auditory and visual facial information while processing language. Here, we ask whether co-verbal visual information provided by talking faces also helps prelexical infants learn the word order of their native language in addition to word frequency and prosodic prominence. We created two structurally ambiguous artificial languages containing head nods produced by an animated avatar, aligned or misaligned with the frequency-based and prosodic information. During 4 minutes, two groups of 4- and 8-month-old infants were familiarized with the artificial language containing aligned auditory and visual cues, while two further groups were exposed to the misaligned language. Using a modified Headturn Preference Procedure, we tested infants’ preference for test items exhibiting the word order of the native language, French, vs. the opposite word order. At 4 months, infants had no preference, suggesting that 4-month-olds were not able to integrate the three available cues, or had not yet built a representation of word order. By contrast, 8-month-olds showed no preference when auditory and visual cues were aligned and a preference for the native word order when visual cues were misaligned. These results imply that infants at this age start to integrate the co-verbal visual and auditory cues.

## Introduction

Discovering the word order of the native language is one of the challenges that infants face during acquisition. Infants tend to follow the word order rules of the target language from their first multiword utterances [1], which suggests that infants configure the basic word order very early in development. What cues allow infants to accomplish this major task? Here, we investigate whether prelexical infants can use co-verbal visual information provided by talking faces, combined with prosody, as a bootstrapping cue to basic word order.

Bootstrapping accounts of language acquisition propose that the input contains perceptually available cues, including distributional and prosodic information, which might allow prelexical infants to discover abstract, perceptually unavailable properties of the target language, supporting the acquisition of syntax [2–3]. Adults and infants have indeed been shown to be sensitive to these distributional and prosodic cues and appear to use them to learn basic properties of their native grammar such as word order [4–6]. For instance, word frequency and the acoustic realization of prosodic prominence allow infants to acquire a rudimentary, but abstract representation of the target language’s basic word order, given the systematic correlation in natural languages between basic word order, on the one hand, and the frequency distribution of functors and content words (distributional cue) and the acoustic realization of phrasal prominence (prosodic cue), on the other hand. Here, we seek to extend these findings, asking how another cue, visual information in talking faces, may contribute to the early bootstrapping of word order.

### Word frequency and phrasal prosody bootstrap basic word order

Functors are few but extremely frequent elements that signal grammatical relations (e.g., determiners, pronouns, verbal inflection: *the*, *he*, *walk-**ed*), whereas content words are numerous, occur much less frequently, and carry lexical meaning (nouns, verbs, adjectives: *turtle*, *walk*, *slow*). In functor-initial languages such as English or Spanish, functors typically occur at the beginning of phrases. This correlates with a series of other word order phenomena such that in these languages, Verbs precede their Objects (VO), they use prepositions (English: *of the woman*), etc. By contrast, in functor-final languages such as Japanese or Basque, where function words appear at the ends of morphosyntactic units, Objects precede Verbs (OV), they use postpositions (Basque: *emakume-a-ren*—woman-the-possessive), etc.

Importantly, infants are sensitive to the frequency and relative order of functors and content words before having lexical knowledge. Gervain and colleagues [5] presented 8-month-old infants with artificial languages that contained strictly alternating frequent and infrequent elements— mirroring functors and content words—and which, crucially, were structurally ambiguous. Infants preferred sequences taken from the stream that followed the order of functors and content words in their native language: 8-month-old learners of Japanese (OV) preferred sequences with a frequent word final order, whereas Italian-learning (VO) 8-month-olds showed the opposite word order preference.

The location and realization of prosodic prominence within phrases also correlate with word order. Phrasal prominence falls on the phrase’s content word [7]. In VO languages, it is realized as a durational contrast, where the stressed vowel of the prominent content word is longer than the functor’s vowel, resulting in a prominence-final, short-long pattern (e.g., English: *to* ***Ro*:***me*). In OV languages, prominence is realized as a pitch (and/or intensity) contrast, where the prominent content word has higher pitch (and/or intensity) than the functor, resulting in a prominence-initial, high-low or strong-weak pattern (e.g., Japanese: ‘***To****kyo ni—*Tokyo in). Prelexical infants are sensitive to this prosodic cue, which they use to parse new input [6,8]. When presented with an ambiguous artificial language similar to the one used by Gervain and colleagues [5], but that additionally contained a durational contrast (i.e., VO prosody), 7-month-old OV-VO bilinguals (e.g., Japanese-English bilinguals) parsed the artificial language into a frequent-initial order, but parsed the language into a frequent-final order if it contained a pitch contrast (i.e., OV prosody) [6]. Indeed, this prosodic cue is proposed to play a crucial role in the acquisition of basic word order in OV-VO bilinguals, that is, infants exposed to phrases with both a frequent-initial order (in their VO language) and a frequent-final order (in their OV language).

### Can visual information cue word order?

Distributional and prosodic information at the phrasal level constitute therefore cues to infants that might help them build a rudimentary representation of the basic word order of the language under acquisition. Importantly, speech perception is inherently multisensory, involving not only the ears but also the eyes, and adults and infants readily integrate auditory and visual facial information while processing language, from the earliest stages of development [9–11]. Phrasal prosody has visual correlates [12]. Here, we therefore investigate whether prelexical infants are able to use facial visual information in addition to word frequency and auditory prosody, as a cue to word order [13].

The visual information that correlates with auditory prosody is co-verbal facial (or peri-oral) gestures such as head and eyebrow motion. These gestures co-occur with speech but do not directly result from the production of speech sounds. Importantly, they correlate with speech acoustics and influence speech perception. Head and eyebrow motion correlate with changes in F0 [14–15] and head motion also correlates with changes in amplitude [16]. Research on co-verbal gestures has to date focused on adult speech perception, and particularly on the link between co-verbal gestures and prosodic prominence. Both head and eyebrow movements have been shown to enhance speech intelligibility in adults [15,17], as well as adults’ perception of prosodic prominence and focus (i.e., the new or contrastive information within an utterance), and hinder perception of prominence when visual and auditory prominence are incongruent [18]. Head and eyebrow movements can even change the realization of acoustic prominence [19–22]. Further, adult speakers signal the boundaries of phrases by means of combined eyebrow movements and head nods [12], and adults can use head nods to parse linguistic input [23].

In sum, co-verbal gestures impact the perception of auditory speech in adults. Here, we ask whether infants rely on this visual prosody, together with word frequency and auditory prosody, to determine word order. We chose to use head movements as markers of visual prosody, as they seem to have an advantage in signaling prominence over eyebrow movements, the other co-verbal gesture associated with visual prosody [20,24].

Recently, we have shown that adults can use head nods in a similar artificial language to make a word order choice [23]. We, therefore, predict that this cue will also be informative for infants to select the word order relevant for their native language in structurally ambiguous artificial languages similar to the ones used in the previous infant and adult studies. We used visual prosody in combination with auditory prosody and word frequency to determine the relative weight of these cues. Specifically, we presented 2 groups of 4-month-old and 2 groups of 8-month-old monolingual learners of English (VO language) with artificial languages containing aligned and misaligned distributional, auditory prosodic and visual cues.

One group of 4-month-old and one group of 8-month-old monolinguals were presented with artificial languages in which aligned VO phrasal prosody (a durational contrast) and visual information (an avatar producing head nods) were overlaid on the frequency-based ambiguous basic structure of alternating frequent and infrequent words. If infants can integrate these aligned audiovisual (AV) cues, as found in adults [23], a frequent-initial parse of the language is expected to obtain in the group of 8-month-olds, an age at which sensitivity to both frequency-based cues and VO prosody has been attested [5–6,8], as well as the ability to integrate different types of distributional and prosodic information, including frequency-based cues and phrasal prosody [2–3]. The absence of a segmentation preference would instead suggest that adding a third source of information to the already present distributional and prosodic cues is too complex for infants to process. The integration of frequency-based and phrasal prosodic cues has to date not been examined in infants younger than 6 months. However, Morgan and Saffran [25] showed that, at 6 months of age, infants fail to integrate correlated sequential and rhythmic information. It is therefore possible that 4-month-olds will not be able to integrate these sources of information. Alternatively, the high redundancy provided by the three aligned cues (frequency, prosody and visual information) might allow even such young infants to segment the ambiguous stream. Therefore, no prediction is drawn for the group of 4-month-olds.

In order to disentangle the relative contributions of the acoustic (frequency and prosodic) and visual cues, a group of 4- and a group of 8-month-old monolinguals were presented with artificial languages that contained aligned distributional and prosodic information, but with the visual cues now misaligned, i.e., with head nods falling on the frequent and prosodically non-prominent element. If the misaligned visual cues are outranked by the distributional and prosodic cues, a frequent-initial segmentation is expected, potentially smaller in magnitude than the one observed in the aligned condition. The absence of a word order preference would alternatively suggest either that the conflicting auditory and visual cues are both processed and equally weighed by the infants, or that the three cues are too complex to process together. In this latter case, a similar absence of a preference would be expected in the aligned and misaligned conditions. Finally, a frequent-final segmentation would indicate that the visual information outranked the aligned distributional and prosodic information. Comparing the parsing preferences of 4- and 8-month-old infants will inform us of potential developmental changes in the relative weight of the auditory and visual facial cues.

## Method

### Participants

Infants were recruited from the Infant Studies Centre’s (University of British Columbia) participant database of infants born in the Greater Vancouver area (Canada). Only families with infants exposed to English at least 80% of the time (i.e., English monolinguals) were invited to participate. A total of 186 infants participated in the experiment. Of those, 50 were 4-month-olds tested in the Aligned condition (i.e., were presented with aligned visual facial and prosodic cues). Twenty of these infants were excluded from analysis due to: crying and fussiness (11), experimenter error (3), equipment failure (3), and parental interference (3). The remaining 30 infants were entered into analysis. A second group of 57 4-month-old infants participated in the Misaligned condition. Of these, 27 infants were excluded from analysis due to: crying and fussiness (20), experimenter error (1), parental interference (4), and not having the minimum number of trials required to be entered into analysis (2). The remaining 30 infants were entered into analysis. Similarly, a group of 40 8-month-old infants took part in the Aligned condition. Thirty of these infants entered analysis, while the remaining 10 infants were excluded due to: crying and fussiness (5), equipment failure (3), and parental interference (2). A fourth group of 39 infants, 8 months of age participated in the Misaligned condition. Thirty infants were entered into analysis. The remaining 9 infants were excluded from analysis due to: crying and fussiness (6), and parental interference (3). Demographic information of the participants is summarized in Table 1. Parents gave informed written consent before participation. The study was approved by the Behavioural Research Ethics Board of the University of British Columbia.

**Table 1:** Demographic information of the infant participants.

	sample	mean age	age range	sex
**4-month-olds aligned**	30	4.01	3.17–4.13	13f, 17m
**4-month-olds misaligned**	30	4.01	3.17–4.16	16f, 14m
**8-month-olds aligned**	30	8.00	7.15–8.17	12f, 18m
**8-month-olds misaligned**	30	8.00	7.15–8.15	14f, 16m

The table includes mean age and age range in months, and sex count in each of the four groups.

### Materials

#### Aligned condition

**Fig 1:**
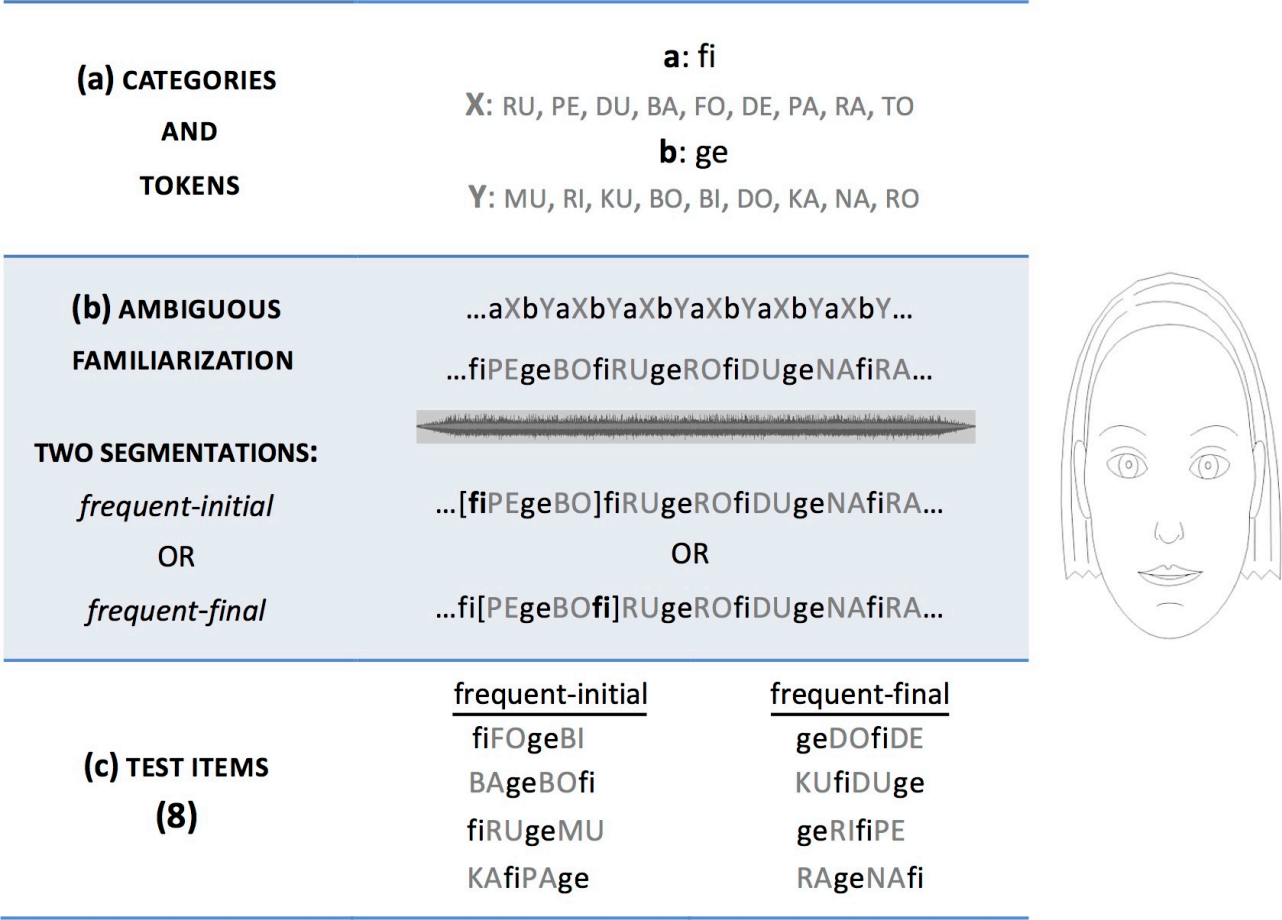
Shared structure of the two artificial languages. The table represents the basic shared structure of the two ambiguous artificial languages: (a) the categories and tokens of the languages, (b) the two possible parses of the ambiguous stream, (c) the 8 test items. On the right, a picture of the animated line drawing.

In the Aligned condition, an ambiguous artificial language was created, based on [4], which consisted of the concatenation of a basic four-syllable-long unit *aXbY* (see Fig 1). Categories *a* and *b* consisted of a single C(onsonant) V(owel) monosyllabic token each, whereas categories *X* and *Y* consisted of 9 different monosyllabic CV tokens each. The tokens in categories *X* and *Y* were therefore 9 times less frequent than the tokens in *a* and *b*. These two categories mimicked the relative frequency of functors (i.e., frequent elements) and content words (i.e., infrequent elements) in natural languages. The basic *aXbY* unit was concatenated 243 times without pauses into a familiarization stream of strictly alternating frequent and infrequent elements, creating a 4 min 17 s long stream minute-long familiarization stream. Phase information was suppressed by ramping the amplitude the initial and final 15 seconds of the stream. The resulting ambiguous stream allowed two possible parses: (i) a frequent-initial (FI) order (*aXbY*), or (ii) a frequent-final (IF) order (*XbYa*).

**Fig 2:**
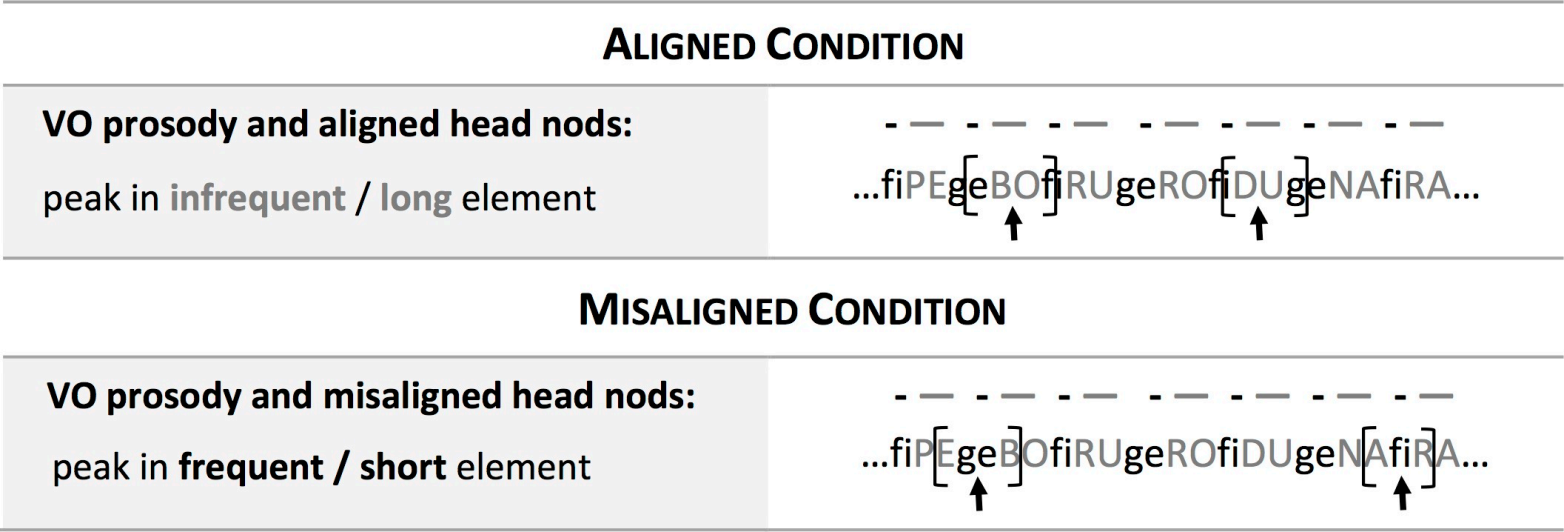
Graphical depiction of the alignment of prosodic and visual information in the aligned and misaligned conditions. The brackets signal the duration of the head nods, while the arrows depict the location of their peak.

The familiarization stream was synthesized using the fr4 female voice of MBROLA [26] at a constant pitch of 200 Hz. The infrequent elements received prosodic prominence, as in natural languages. Thus, the infrequent elements were longer (144 ms per segment) than the non-prominent frequent elements (120 ms per segment). In addition to this contrast in duration, the artificial language also contained visual information: an animated line drawing of a female face (Blender, version 2.75; see Fig 1), which produced head nods. These nods resulted from the combination of two distortions: an increase in head size and a rotation forward of the head with the axis on the drawing’s chin. Each nod had a duration of 480 ms, consisting of a stroke phase of 240 ms and a retraction phase of 240 ms. The drawing’s mouth opened and closed gradually as a function of the stream’s amplitude, to increase the perceived naturalness of the avatar without providing detailed segmental information. The peak of the head nods occurred at the center of the infrequent and prosodically prominent syllable, providing aligned visual and prosodic information about prominence. As speakers do not produce regularly timed nods, a total of 191 nods were assigned to pseudorandom locations in the stream according to the following criteria (see Fig 2): consecutive nods were separated by a minimum of four syllables (i.e., the length of the basic *aXbY* unit), both infrequent categories had a similar number of nods (i.e., 96 nods fell on category *X* and 95 on category *Y*), and no more than three consecutive nods fell on the same category.

Test items were eight four-syllable-long items, presented only auditorily and with no prosodic cues. Four instantiated a frequent-initial order (*aXbY*: e.g., *fiFOgeBI*), while the other four had a frequent-final order (*XbYa*: e.g., *KAfiPAge*). They were also synthesized with the fr4 Mbrola voice at 200 Hz and had equal syllable durations (120 ms per segment). Each test trial consisted of 15 repetitions of a single test item, separated by 500 ms pauses and presented only auditorily. No more than two trials of the same word order type (frequent-initial vs. frequent-final) occurred consecutively.

#### Misaligned condition

The artificial language used in this condition differed from the language in the Aligned condition in a single feature, namely, the alignment of the head nods. The peak of the head nods occurred at the center of the frequent and prosodically non-prominent syllable, which therefore resulted in misaligned visual and prosodic information about prominence. In all other respects, the artificial language, familiarization stream and test items were identical to the ones described in the Aligned condition.

### Procedure

**Fig 3:**
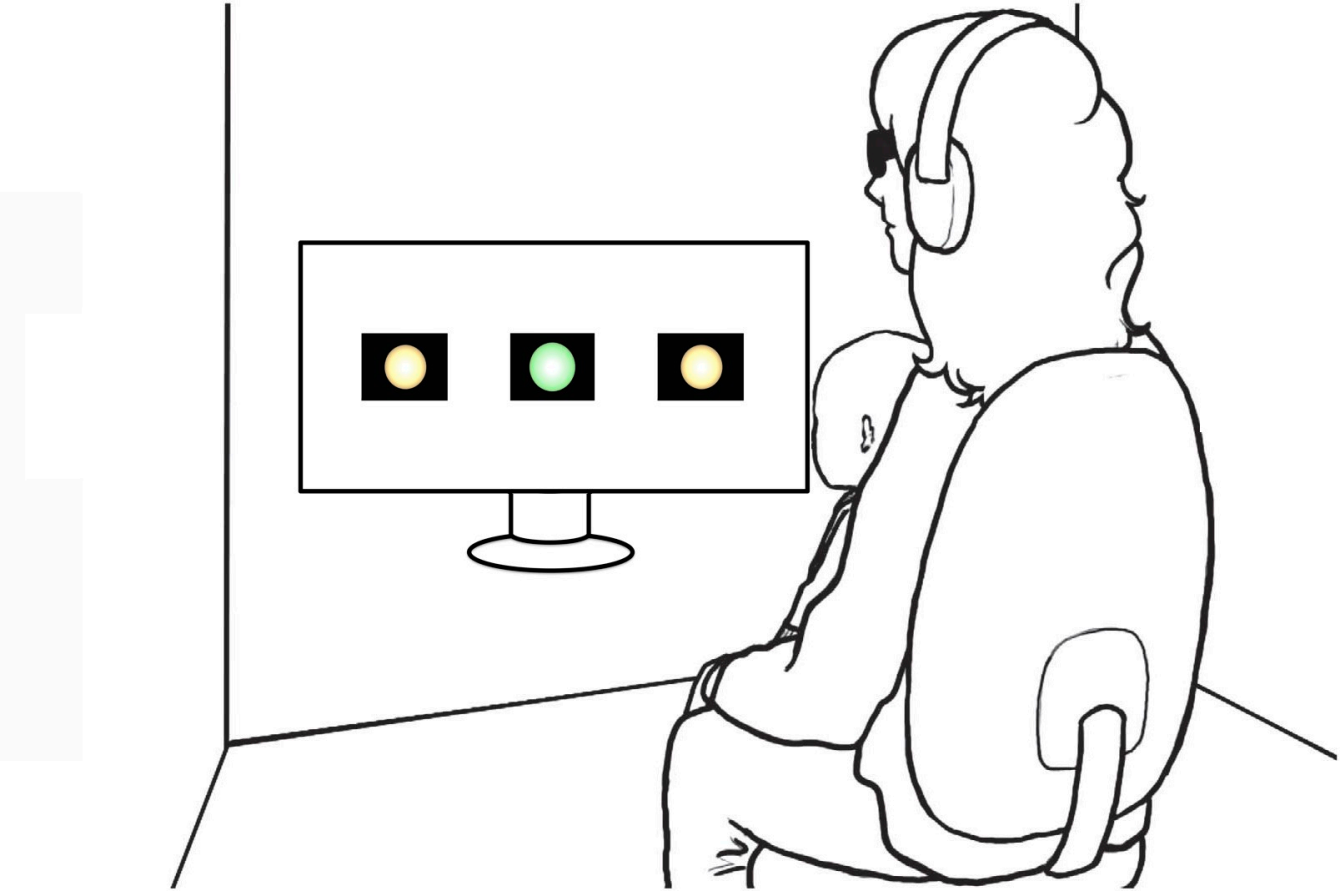
Graphical depiction of the study’s modified HPP (image adapted from [6]). The size of the lights as they appear on the drawing of the screen is scaled to the actual size displayed on the 46” screen during the study.

The study took place at the Infant Studies Centre (UBC), in Vancouver, Canada. To test infants’ segmentation preferences, we used a modified version of the headturn preference paradigm (HPP), adapted to display the stimuli in a single wide screen (46” LCD monitor, see Fig 3). In the classic HPP [27], a central fixation light is placed in front of the caregiver and infant, and another two fixation lights are placed on the sides of the room. In the current setup, however, the central and sidelights were presented as videos displayed on a single wide screen. This modification was necessary to be able to project the visual information, i.e. the video of the avatar. Such a setup was successfully used by González-Gómez and colleagues [28]. Each of the lights occupied 200 pixels, that is, 16.6% of the screen, and the outer edge of the sidelights laid 160 pixels, i.e., 13.2%, from the outer edge of the screen. Infants were seated on a parent’s lap in a sound-attenuated room with dim lights. A webcam was placed below the screen and recorded the infant’s looking behavior. In order to prevent parental influence on the infants’ behavior, caregivers listened to masking music over headphones. An experimenter wearing headphones with masking music monitored infants’ looking behavior and controlled the lights and the stimuli.

Stimuli were displayed using PsyScope software [29]. The study started with an attention getter— a bouncing ball—in order to capture the infant’s attention, after which familiarization began. During familiarization, the 4-minute-long video of the audiovisual speech stream was played, continuously, on the screen. The avatar appeared at the center of the screen and had roughly the size of the head of a human adult. Throughout familiarization, the avatar appeared to nod as in Fig 2 above (see the Supporting Information for short clips of the familiarization in the aligned and misaligned conditions). Immediately after familiarization, infants were tested for their segmentation preference in sixteen auditory-only (no avatar) test trials. Each trial began with a green blinking central light that aimed to attract the infant’s attention. Once the infant fixated centrally, this light disappeared and one of the yellow sidelights appeared on screen. Side of presentation was counterbalanced across test trials and babies. When the infant fixated on the blinking sidelight, the experimenter started playing a test stimulus, which continued until its end (22 s) or until the infant looked away for more than 2 seconds. After this, a new trial began. The session was videotaped.

## Results

The infants’ looking behavior during test was coded and measured off-line. We applied the same exclusion criteria as Gervain and colleagues [5–6]. All trials were excluded from analysis that had looking times less than 960 ms, that is, the duration of a single test item. Similarly, trials with looking times that exceeded the maximum duration of the trial, 22 seconds, were excluded. After applying these criteria, only infants that had a minimum of three frequent-initial and three frequent-final trials in the first eight trials were retained for analysis. In total, 120 babies—30 in each group—were entered into the analysis (the full set of data are available in the Supporting Information). We conducted a power analysis taking Gervain and Werker [6] as reference, as they tested English monolingual 7-month-olds with a similar artificial language that contained frequency-based cues (dz = 0.53). The analysis revealed that adequate power (0.8) could be obtained from a sample size of n = 30 per group.

**Fig 4:**
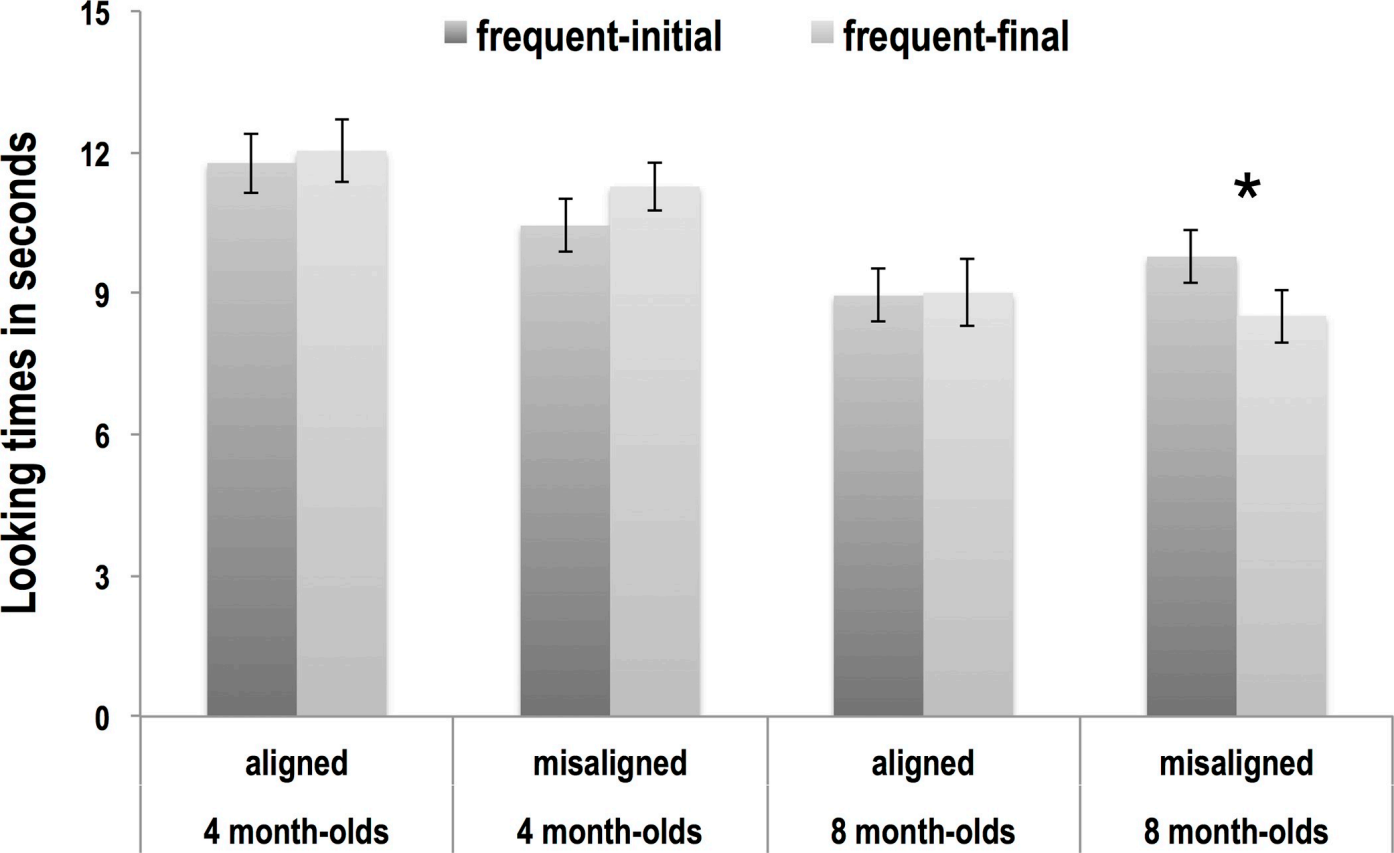
Looking time results. The x axis shows the four groups examined. The y axis displays the infants’ looking times in seconds. The dark grey bars depict looking times to the frequent-initial items, and the light grey bars looking times to the frequent-final items. Error bars represent the standard error of the mean.

The infants’ looking times were averaged across all trials of the same word order (frequent-initial or frequent-final) (see Fig 4 and Table 2). Analyses were conducted using DataDesk software. A repeated-measures ANOVA was carried out with looking time as the dependent variable, Age (4 vs. 8 months) and Type of AV Information (aligned vs. misaligned) as between-subject variables, and Order (frequent-initial vs. frequent-final) as the within-subject variable. The ANOVA yielded a significant fixed effect of Age (F(1,118) = 17,541, *p* < .0001, partial ŋ^2^ = 0.411 due to longer overall looking times in 4-month-olds than in 8-month-olds, a significant interaction between Order and Age (F(1,118) = 5.194, *p* = .025, partial ŋ^2^ = .042), and a marginally significant interaction between Order, Age, and Type of AV information (F(1,118) = 3.514, *p* = .063, partial ŋ^2^ = .029). To further explore these interactions, we compared looking times to the two orders within groups using pair-wise Scheffé post-hoc tests. Neither group of 4-month-olds, nor the group of 8-month-olds presented with aligned AV information displayed a preference (all *p* ≥ .102), that is, they looked equally long to the frequent-initial and frequent-final items. However, the group of 8-month-olds exposed to misaligned AV information had a significant preference—i.e., longer looking times—for the frequent-initial test items (*p* = .014, *d* = .410).

**Table 2:** Looking time results.

	frequent-initial trials		frequent-final trials	
	mean	SE	mean	SE
**4-month-olds aligned**	11.77	0.63	12.03	0.67
**4-month-olds misaligned**	10.44	0.56	11.27	0.51
**8-month-olds aligned**	8.95	0.56	9.01	0.70
**8-month-olds misaligned**	9.78	0.56	8.52	0.57

The table depicts mean looking times in seconds and standard error of the mean (SE) obtained in each of the four groups of infants in frequent-initial and frequent-final trials.

## Discussion

The present study investigated the abilities of prelexical infants to integrate frequency-based, prosodic and co-verbal visual information to parse speech. Specifically, we examined whether the presence of head nods modulated prelexical infants’ parsing of the input, when aligned or misaligned with auditory information. Four- and 8-month-old infants were presented with structurally ambiguous artificial languages that contained frequency-based information (alternating frequent and infrequent elements), the phrasal prosodic pattern characteristic of English, the VO language they were acquiring (a durational contrast), and head nods produced by an animated avatar. Auditory and visual information were either aligned, with the nod peaking over the prosodically prominent element, or were misaligned, with the nod peaking at the prosodically non-prominent element. We thus aimed to determine the relative weight of the congruent and incongruent auditory and visual cues at two points of development (4- and 8-months of age).

Analysis revealed a significant effect of age, an interaction between the age of the infants and the word order of the test items (frequent-initial vs. frequent-final), and a trend towards a significant interaction between age, order, and the type of audiovisual information provided (aligned vs. misaligned audiovisual cues). These interactions reveal differing looking patterns in 4- and 8-month-olds, which were further explored separately in the four groups.

The two groups of 4-month-olds showed no segmentation preference. The alignment of the auditory and visual information did therefore not modulate their looking patterns. This absence of a preference might result from the infants being too young to be able to process and integrate the available sources of information, despite their high redundancy. Alternatively or in addition, they might not have built a representation of the target language’s word order yet. The present results with 4-month-olds suggest that the specific distributional information provided by the frequency and relative order of functors and content words might be acquired later in development, and/or that the presence of redundant auditory and visual cues does not assist infants this age.

Our results with the 8-month-old infants, by contrast, evidenced a word order preference, but only in the misaligned condition. This result is surprising. Eight-month-old infants are sensitive to frequency-based information and phrasal prosody, which they integrate to parse ambiguous streams similar to the one used in the present study [6,8]. At this age, infants are also able to detect whether or not there is congruence between the auditory and visual (phonetic) signals in an unfamiliar language [30], and can use visual information (both visual speech and an oscilloscope pattern) to segment words from speech as long as it is synchronized with the auditory signal [31]. A frequent-initial segmentation preference was thus predicted in the group of 8-month-olds presented with aligned auditory and visual information. Contrary to prediction, infants, unlike adults [23] and 8-month-olds presented with auditory-only cues [6,8], showed no segmentation preference in this condition. Interestingly, however, infants in the misaligned condition showed a preference towards frequent-initial segmentation. The absence of a preference observed in the aligned condition suggests that the 8-month-olds attempted to integrate the available AV cues. Had infants disregarded visual information altogether, a frequent-initial segmentation preference—signaled by the two auditory cues—would have been observed, similarly to the one found in the infants presented with misaligned AV cues. We put forward three, not mutually incompatible interpretations for this pattern of results, to be pursued in future research.

First, this pattern could obtain if infants had perceived the misaligned visual cues as being aligned, and the aligned ones as misaligned. This misinterpretation of the cues could potentially result from a particular characteristic of the current study’s audiovisual streams, namely the duration of the head nods relative to the duration of the CV tokens. In the artificial languages, frequent elements were 240 ms long, whereas infrequent elements— which also were prosodically prominent—were 288 ms long. The head nods produced by the animated avatar had a duration of 480 ms, consisting of a 240 ms stroke phase and a 240 ms retraction phase. In the stream with aligned cues, the peak of the nods occurred at the center of the 288 ms long target token. Consequently, a portion (96 ms) of the stroke and the retraction took place in the preceding and subsequent elements, respectively. Perfect synchrony is not required for infants to integrate auditory and visual information. In fact, 2- to 8-month-olds can accommodate up to 300 ms of temporal separation between the auditory and visual signals before perceiving them as separate [32]. However, head nods appear to boost perceived prominence most when aligned with the right boundary of a (monosyllabic) word [22], and naturally produced nods tend to appear in the target word but often occur in the post-target word, too [33]. Thus, the location of the nod’s peak might not necessarily align with the location of acoustic prominence. The short duration of the current study’s tokens (240–288 ms) and the fact that nods spanned three tokens (the target token and the two surrounding ones) might thus have driven infants to misinterpret the alignment of the visual cue.

Additionally, the movements of the avatar’s mouth might have added further complexity to the task. While the mouth moved as a function of the amplitude of the stream, it did not provide reliable articulatory information. As young as 2 months of age, infants match auditory vowels to the corresponding silent talking face [34–35], and at 4.5 months of age infants can match auditory and visual speech presented sequentially—with no temporal synchrony cues—even in an unfamiliar language [36]. As at 8 months of age infants’ attention is preferentially directed to the talker’s mouth [37], the lack of exact correspondence between auditory and visual speech could potentially have impacted infants’ processing of the stream, maybe directing their attention to the avatar’ mouth. Note, however, that Shaw and colleagues [38] report that prelexical infants’ (5–10 months) looking times to talking faces are not modulated by the presence of incongruent auditory and visual speech (e.g., native auditory speech paired with non-native visual speech, or vice versa).

An artificial language where nods occur within single tokens and where the avatar’s mouth provides more detailed information would confirm or rule out this first hypothesis.

Second, the pattern of results obtained might reflect a limited ability to make use of co-verbal facial gestures, as compared with the infants’ sophisticated abilities to process visual speech. Prelexical infants take advantage of the tight coupling between visual speech and the speech acoustics [16] and make fine-grained use of visual speech [34–40]. Co-verbal facial gestures such as head nods also correlate with speech acoustics. Relevant for the present study, head motion has been shown to correlate with changes in F0 [15] and amplitude [16]. However, these co-verbal gestures have great inter- and intra-speaker variability [41] and, unlike visual speech, are not causally related to auditory speech. Therefore, these gestures are less reliable cues than visual speech. A recent study that analyzed the presence of head nods in elicited speech in Japanese and English, observed that 31–55% of the utterances contained no nods within target phrases, depending on the language and speech style [12]. Furthermore, the use and frequency of co-verbal gestures appears to be modulated by factors such as the strength of the prominent element or the phrasing of the utterance: in Japanese, head nods are found in about 30–40% of phrases containing strong boundaries, but only in 10–15% with weak boundaries [42], and English talkers mark phrasal stress using head and eyebrow movements but word stress is only marked with head motion [43]. In the current study, only 20% of the elements heard during familiarization were accompanied by head nods (i.e., 191 of 972 tokens) that were interspersed, as natural head nods do not occur periodically.

Thiessen reports that, unlike adults, 8-month-olds also do not benefit from the presence of shapes synchronized with “words” to segment an artificial language, even when each shape is paired with a single “word”, creating a sort of word-object relation [44]. He argues that such young infants might not have accumulated sufficient experience with word-object relations to benefit from them. It is therefore possible that 8-month-old infants are also still discovering the relation between head nods and prosodic prominence—given their rather unsystematic co-occurrence—and make limited use of this co-verbal gesture. At 9 months of age, infants can detect whether word prominence aligns or not with the stroke of a pointing hand gesture [45]. Nine-month-old infants might similarly be able to detect the misalignment of head nods. Further, given that adult speakers spontaneously produce eyebrow movements accompanied by head nods at phrase boundaries [12], infants could benefit to a greater extent from the combination of these two visual cues to parse phrases from new input, presumably as a result of their greater perceptual salience.

Third, the present pattern of results could be interpreted as suggesting that, at 8-months, infants tried to integrate the co-verbal visual and auditory cues but failed. The observed absence of a preference in the aligned condition could be interpreted as suggesting that adding a third source of information to the two auditory cues resulted in a signal too complex for such young infants to process. In turn, the almost significant frequent-initial preference observed when exposed to misaligned cues could suggest that infants detected the incongruence between the auditory and visual facial cues (or at least were unable to integrate the facial cues in any way), and hence ignored the unreliable visual information and segmented the language based on the cumulative and more stable auditory sources of information, as found in certain instances with adults [46–47]. This interpretation would entail that, while aligned visual facial gestures can facilitate segmentation in adults, their presence presents an information overload for infants who are just at the cusp of being able to integrate frequency and prosodic acoustic information.

Although we cannot rule out this last interpretation, we consider it to be unlikely that complexity may be an issue. In day-to-day life, infants are regularly exposed to multiple auditory and visual cues, e.g., the talking faces of their parents and other members of the household, other children and caretakers in day care, etc. Given the infants’ abovementioned refined abilities to process and integrate auditory and visual speech, attested from early stages of development [34–40], simultaneous exposure to auditory and co-verbal visual cues is unlikely to disrupt infants’ learning because of its complexity. It is, however, still possible that reasons other than complexity,—e.g., some of the factors discussed above or any other reason—prevented infants from appropriately integrating the visual information. An unintegrated and hence uninformative cue might be more easily ignored when misaligned than when it is aligned. Future work is needed in order to understand the trajectory of the infants’ ability to process co-verbal facial gestures.

## Conclusions

In a study with four groups of English-learning infants, we explored whether infants at 4- and 8-months of age use co-verbal visual gestures, specifically head nods, to parse an artificial language into phrase-like units. Infants were presented with structurally ambiguous languages, in which an animated avatar produced head nods that were either aligned or misaligned with frequency-based and prosodic cues. At 4 months, infants are not able to use the three available cues. This result suggests that infants this young have yet to acquire the specific properties of the native language necessary for implementing these parsing strategies. At 8 months, infants’ abilities to use co-verbal gestures seem to be emerging, but are still limited. Further work is necessary to understand infants’ capacity to process concurrent auditory and co-verbal visual prosody.

## Supporting information

S1 Excel file Full set of data. The excel file contains participants’ mean looking times in frequent-initial and frequent-final test trials, and their respective number of trials. The document contains 4 sheets, one per each of the groups tested.
(XLSX)

S1 Quicktime video movie Familiarization demo: Aligned condition. The video contains a 10 second clip from the familiarization presented in the aligned condition.
(MOV)

S2 Quicktime video movie Familiarization demo: Misaligned condition. The video contains a 10 second clip from the familiarization presented in the misaligned condition.
(MOV)
